# Poly[bis­(methanol-κ*O*)tris­(μ-pyrimidine-κ^2^ *N*:*N*′)tetra­kis(thio­cyanato-κ*N*)dinickel(II)]

**DOI:** 10.1107/S1600536809005674

**Published:** 2009-03-06

**Authors:** Mario Wriedt, Sina Sellmer, Inke Jess, Christian Näther

**Affiliations:** a Institut für Anorganische Chemie, Christian-Albrechts-Universität Kiel, Max-Eyth-Strasse 2, D-24118 Kiel, Germany

## Abstract

In the crystal structure of the title compound, [Ni_2_(NCS)_4_(C_4_H_4_N_2_)_3_(CH_3_OH)_2_]_*n*_, each nickel(II) cation is coordinated by three *N*-bonded pyrimidine ligands, two *N*-bonded thio­cyanate anions and one *O*-bonded methanol mol­ecule in a distorted octa­hedral environment. The asymmetric unit consists of one nickel cation, two thio­cyanate anions and one methanol mol­ecule in general positions, as well as one pyrimidine ligand located around a twofold rotation axis. The crystal structure consists of μ-*N*:*N*′ pyrimidine-bridged zigzag-like nickel thio­cyanate chains; these are further linked by μ-*N*:*N*-bridging pyrimidine ligands into layers which are stacked perpendicular to the *b* axis. The layers are connected via weak O—H⋯S hydrogen bonding.

## Related literature

For related pyrimidine structures, see: Lloret *et al.* (1998 ▶); Näther *et al.* (2007 ▶); Näther & Greve (2003 ▶). For general background, see: Wriedt *et al.* (2008 ▶) and literature cited therein.
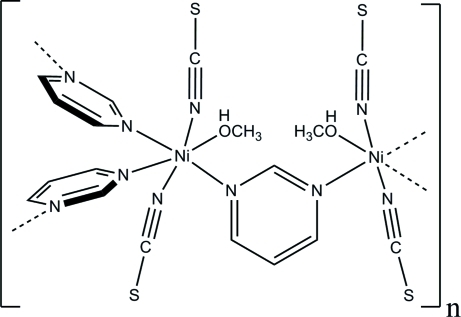


## Experimental

### 

#### Crystal data

[Ni_2_(NCS)_4_(C_4_H_4_N_2_)_3_(CH_4_O)_2_]
*M* *_r_* = 654.10
Orthorhombic, 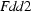

*a* = 20.0624 (4) Å
*b* = 32.5018 (6) Å
*c* = 8.0268 (2) Å
*V* = 5233.99 (19) Å^3^
*Z* = 8
Mo *K*α radiation
μ = 1.80 mm^−1^
*T* = 80 K
0.19 × 0.09 × 0.03 mm

#### Data collection

Stoe IPDS-2 diffractometer
Absorption correction: numerical (*X-SHAPE* and *X-RED32*; Stoe & Cie, 2008 ▶) *T*_min_ = 0.813, *T*_max_ = 0.936
26228 measured reflections
3743 independent reflections
3659 reflections with *I* > 2σ(*I*)
*R*_int_ = 0.043

#### Refinement

*R*[*F* ^2^ > 2σ(*F* ^2^)] = 0.020
*wR*(*F* ^2^) = 0.048
*S* = 1.09
3743 reflections
170 parameters
1 restraint
H atoms treated by a mixture of independent and constrained refinement
Δρ_max_ = 0.34 e Å^−3^
Δρ_min_ = −0.21 e Å^−3^
Absolute structure: Flack (1983 ▶), 1746 Friedel pairs
Flack parameter: 0.092 (7)

### 

Data collection: *X-AREA* (Stoe & Cie, 2008 ▶); cell refinement: *X-AREA*; data reduction: *X-RED32* (Stoe & Cie, 2008 ▶; program(s) used to solve structure: *SHELXS97* (Sheldrick, 2008 ▶); program(s) used to refine structure: *SHELXL97* (Sheldrick, 2008 ▶); molecular graphics: *XP* in *SHELXTL* (Sheldrick, 2008 ▶); software used to prepare material for publication: *XCIF* in *SHELXTL*.

## Supplementary Material

Crystal structure: contains datablocks I, global. DOI: 10.1107/S1600536809005674/si2156sup1.cif

Structure factors: contains datablocks I. DOI: 10.1107/S1600536809005674/si2156Isup2.hkl

Additional supplementary materials:  crystallographic information; 3D view; checkCIF report

## Figures and Tables

**Table 1:** Selected geometric parameters (Å, °)

Ni1—N31	2.0334 (14)
Ni1—N21	2.0376 (13)
Ni1—O41	2.1053 (12)
Ni1—N11	2.1118 (13)
Ni1—N2^i^	2.1200 (13)
Ni1—N1	2.1244 (13)

Symmetry code: (i) 
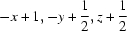
.

**Table 2:** Hydrogen-bond geometry (Å, °)

*D*—H⋯*A*	*D*—H	H⋯*A*	*D*⋯*A*	*D*—H⋯*A*
O41—H1*O*4⋯S21^ii^	0.80 (3)	2.50 (3)	3.2474 (14)	154 (2)

Symmetry code: (ii) 
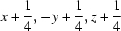
.

**Fig. 1.**
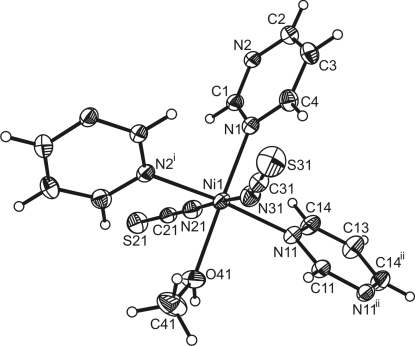
Crystal structure of the title compound with labelling and displacement ellipsoids drawn at the 50% probability level. [Symmetry codes: (i) -x+1, -y+1/2, z+1/2; (ii) -x+3/2, -y+1/2, z.]

**Fig. 2.**
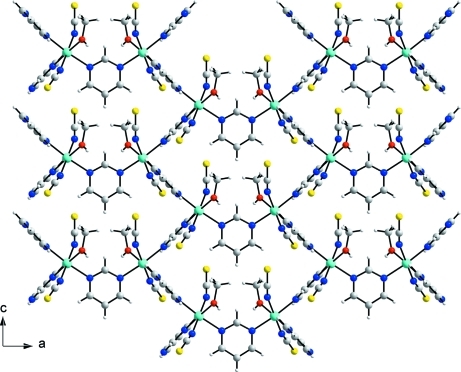
The crystal structure of the title compound with view along the b axis.

**Fig. 3.**
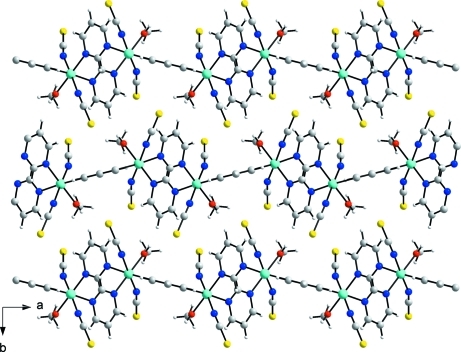
The crystal structure of the title compound with view along the c axis.

## supplementary crystallographic information

### Comment

Recently, we have shown that new ligand deficient coordination polymers with interesting magnetic properties can be prepared by thermal decomposition of suitable ligand rich precursor compounds (Näther & Greve, 2003; Wriedt *et al.*,  2008; and Näther *et al.*, 2007). In our ongoing investigation on the synthesis, structures and properties of new coordination polymers based on paramagnetic metal pseudohalides and N-donor ligands (Lloret *et al.* 1998), we have reacted nickel(II) thiocyanate with pyrimidine in methanol. In this reaction single crystals of the title compound has been formed.

The 2:3 title compound [Ni(SCN)_2_(pyrimidine)_3_*2MeOH]_n_ (Fig. 1) represents a two-dimensional coordination polymer, which consists of µ-1,3-(*N*,*N*) pyrimidine bridged zigzag like nickel thiocyanates chains, which are further linked by µ-1,3-(*N*,*N*) bridged pyrimidine ligands into layers (Fig. 2 and 3). Within each layer the nickel cations are bridged by three µ-1,3-(*N*,*N'*) pyrimidine ligands and are further terminal coordinated by two *N*-bonded thiocyanate anions and one *O*-bonded methanol molecule. Thus, each nickel cation is octahedrally coordinated. The asymmetric unit consists of one nickel cation, two thiocyanate anions and one methanol molecule in general position as well as one pyrimidine ligand located around a twofold rotation axis. The Ni—NCS distances amount to 2.0334 (14) and 2.0376 (13) Å and the Ni—N_pyrimidine_ distances range from 2.1118 (13) to 2.1244 (13) Å as well as the angles around the iron cations range between 86.62 (5) and 177.66 (5)° (Tab. 1). The shortest intra- and interchain Ni···Ni distances amount to 5.9470 (1) and 8.4023 (1) Å, respectively.

### Experimental

Ni(SCN)_2_, pyrimidine and methanol were obtained from Alfa Aesar. 0.125 mmol (21.5 mg) Ni(SCN)_2_, 0.25 mmol (20.0 mg) pyrimidine and 0.5 ml methanol were transfered in a closed test-tube. The mixture was heated at 120 °C for three days. After cooling green needle-shaped single crystals of the title compound were obtained in a heterogenous mixture.

### Refinement

All H atoms were located in difference map but were positioned with idealized geometry and were refined isotropic with *U*_eq_(H) = 1.2 *U*_eq_(C) of the parent atom using a riding model with C—H = 0.95 Å.

The absolute structure was determined on the bases of 1746 Friedel pairs. The crystal was racemically twinned and therefore a twin refinement was performed (BASF: 0.09169 with e.s.d.: 0.00739).

### Crystal data

**Table:**

[Ni_2_(NCS)_4_(C_4_H_4_N_2_)_3_(CH_4_O)_2_]	*F*(000) = 2672
*M**_r_* = 654.10	*D*_x_ = 1.660 Mg m^−^^3^
Orthorhombic, *F**d**d*2	Mo *K*α radiation, λ = 0.71073 Å
Hall symbol: F 2 -2d	Cell parameters from 26829 reflections
*a* = 20.0624 (4) Å	θ = 2.4–30.2°
*b* = 32.5018 (6) Å	µ = 1.80 mm^−^^1^
*c* = 8.0268 (2) Å	*T* = 80 K
*V* = 5233.99 (19) Å^3^	Needle, green
*Z* = 8	0.19 × 0.09 × 0.03 mm

### Data collection

**Table:**

Stoe IPDS-2 diffractometer	3743 independent reflections
Radiation source: fine-focus sealed tube	3659 reflections with *I* > 2σ(*I*)
graphite	*R*_int_ = 0.043
ω scans	θ_max_ = 29.8°, θ_min_ = 2.4°
Absorption correction: numerical (*X-SHAPE* and *X-RED32*; Stoe & Cie, 2008)	*h* = −28→28
*T*_min_ = 0.813, *T*_max_ = 0.936	*k* = −45→45
26228 measured reflections	*l* = −11→11

### Refinement

**Table:**

Refinement on *F*^2^	Secondary atom site location: difference Fourier map
Least-squares matrix: full	Hydrogen site location: inferred from neighbouring sites
*R*[*F*^2^ > 2σ(*F*^2^)] = 0.020	H atoms treated by a mixture of independent and constrained refinement
*wR*(*F*^2^) = 0.048	*w* = 1/[σ^2^(*F*_o_^2^) + (0.0258*P*)^2^ + 3.6623*P*] where *P* = (*F*_o_^2^ + 2*F*_c_^2^)/3
*S* = 1.09	(Δ/σ)_max_ = 0.001
3743 reflections	Δρ_max_ = 0.34 e Å^−^^3^
170 parameters	Δρ_min_ = −0.21 e Å^−^^3^
1 restraint	Absolute structure: Flack (1983), 1746 Friedel pairs
Primary atom site location: structure-invariant direct methods	Flack parameter: 0.0917 (74)

### Special details

**Table:**

Geometry. All e.s.d.'s (except the e.s.d. in the dihedral angle between two l.s. planes) are estimated using the full covariance matrix. The cell e.s.d.'s are taken into account individually in the estimation of e.s.d.'s in distances, angles and torsion angles; correlations between e.s.d.'s in cell parameters are only used when they are defined by crystal symmetry. An approximate (isotropic) treatment of cell e.s.d.'s is used for estimating e.s.d.'s involving l.s. planes.
Refinement. Refinement of *F*^2^ against ALL reflections. The weighted *R*-factor *wR* and goodness of fit *S* are based on *F*^2^, conventional *R*-factors *R* are based on *F*, with *F* set to zero for negative *F*^2^. The threshold expression of *F*^2^ > σ(*F*^2^) is used only for calculating *R*-factors(gt) *etc*. and is not relevant to the choice of reflections for refinement. *R*-factors based on *F*^2^ are statistically about twice as large as those based on *F*, and *R*- factors based on ALL data will be even larger.

### Fractional atomic coordinates and isotropic or equivalent isotropic displacement parameters (Å^2^)

**Table:**

	*x*	*y*	*z*	*U*_iso_*/*U*_eq_	
Ni1	0.606548 (9)	0.227000 (5)	0.78384 (2)	0.01902 (4)	
N1	0.55175 (7)	0.27249 (4)	0.65206 (17)	0.0215 (2)	
N2	0.47332 (7)	0.29025 (4)	0.44204 (17)	0.0223 (2)	
C1	0.50701 (7)	0.26330 (4)	0.5344 (2)	0.0221 (2)	
H1	0.4985	0.2350	0.5149	0.026*	
C2	0.48569 (8)	0.33035 (5)	0.4691 (2)	0.0254 (3)	
H2	0.4634	0.3505	0.4036	0.030*	
C3	0.52997 (9)	0.34281 (5)	0.5898 (2)	0.0270 (3)	
H3	0.5379	0.3712	0.6103	0.032*	
C4	0.56250 (9)	0.31286 (5)	0.6799 (2)	0.0256 (3)	
H4	0.5933	0.3208	0.7638	0.031*	
N11	0.69268 (6)	0.24119 (4)	0.64396 (16)	0.0216 (2)	
C11	0.7500	0.2500	0.7204 (3)	0.0221 (4)	
H11	0.7500	0.2500	0.8388	0.027*	
C13	0.7500	0.2500	0.3882 (3)	0.0322 (5)	
H13	0.7500	0.2500	0.2698	0.039*	
C14	0.69298 (8)	0.24151 (6)	0.4778 (2)	0.0275 (3)	
H14	0.6528	0.2357	0.4196	0.033*	
N21	0.58003 (7)	0.18291 (4)	0.61601 (18)	0.0257 (3)	
C21	0.55990 (7)	0.15388 (4)	0.5486 (2)	0.0223 (3)	
S21	0.53215 (3)	0.113396 (13)	0.45037 (6)	0.03346 (9)	
N31	0.63443 (7)	0.26781 (4)	0.96274 (18)	0.0249 (3)	
C31	0.63460 (8)	0.29113 (5)	1.07238 (19)	0.0228 (3)	
S31	0.63423 (3)	0.323347 (15)	1.22718 (6)	0.03821 (11)	
O41	0.66278 (7)	0.18126 (4)	0.90523 (16)	0.0300 (3)	
C41	0.67557 (12)	0.17680 (6)	1.0780 (3)	0.0418 (5)	
H41A	0.6591	0.2011	1.1375	0.063*	
H41B	0.7237	0.1740	1.0962	0.063*	
H41C	0.6528	0.1522	1.1198	0.063*	
H1O4	0.6830 (14)	0.1640 (8)	0.854 (3)	0.047 (7)*	

### Atomic displacement parameters (Å^2^)

**Table:**

	*U*^11^	*U*^22^	*U*^33^	*U*^12^	*U*^13^	*U*^23^
Ni1	0.01457 (7)	0.02028 (8)	0.02220 (8)	−0.00051 (7)	−0.00029 (7)	−0.00255 (7)
N1	0.0171 (6)	0.0219 (6)	0.0254 (6)	−0.0014 (4)	−0.0012 (4)	0.0006 (5)
N2	0.0169 (6)	0.0217 (5)	0.0282 (6)	−0.0005 (5)	−0.0006 (5)	0.0003 (5)
C1	0.0175 (6)	0.0196 (5)	0.0291 (6)	−0.0006 (4)	−0.0011 (6)	−0.0018 (6)
C2	0.0253 (8)	0.0222 (6)	0.0287 (7)	0.0000 (5)	0.0014 (6)	0.0019 (6)
C3	0.0329 (9)	0.0193 (6)	0.0287 (7)	−0.0039 (6)	0.0002 (6)	−0.0016 (5)
C4	0.0276 (8)	0.0239 (6)	0.0254 (7)	−0.0048 (6)	−0.0021 (6)	−0.0014 (5)
N11	0.0165 (6)	0.0253 (6)	0.0231 (6)	−0.0003 (5)	0.0005 (5)	−0.0011 (4)
C11	0.0173 (9)	0.0262 (9)	0.0230 (9)	0.0002 (7)	0.000	0.000
C13	0.0257 (12)	0.0499 (15)	0.0208 (10)	−0.0044 (10)	0.000	0.000
C14	0.0202 (7)	0.0376 (8)	0.0247 (7)	−0.0016 (6)	−0.0022 (6)	−0.0019 (6)
N21	0.0230 (6)	0.0245 (6)	0.0296 (7)	−0.0003 (5)	−0.0028 (5)	−0.0053 (5)
C21	0.0183 (6)	0.0246 (6)	0.0239 (6)	0.0029 (5)	0.0015 (5)	0.0018 (5)
S21	0.0355 (2)	0.02680 (18)	0.0381 (2)	−0.00436 (16)	−0.00331 (18)	−0.00964 (16)
N31	0.0222 (6)	0.0263 (6)	0.0262 (6)	−0.0024 (5)	0.0006 (5)	−0.0035 (5)
C31	0.0177 (6)	0.0241 (6)	0.0265 (8)	−0.0009 (5)	0.0005 (5)	0.0012 (5)
S31	0.0431 (3)	0.0360 (2)	0.0355 (2)	−0.00220 (19)	0.00501 (19)	−0.01510 (18)
O41	0.0285 (6)	0.0306 (6)	0.0308 (6)	0.0096 (5)	−0.0014 (5)	0.0020 (5)
C41	0.0516 (12)	0.0336 (9)	0.0403 (10)	0.0021 (8)	−0.0217 (9)	0.0030 (7)

### Geometric parameters (Å, °)

**Table:**

Ni1—N31	2.0334 (14)	N11—C14	1.334 (2)
Ni1—N21	2.0376 (13)	N11—C11	1.3346 (16)
Ni1—O41	2.1053 (12)	C11—N11^iii^	1.3346 (16)
Ni1—N11	2.1118 (13)	C11—H11	0.9500
Ni1—N2^i^	2.1200 (13)	C13—C14	1.379 (2)
Ni1—N1	2.1244 (13)	C13—C14^iii^	1.379 (2)
N1—C1	1.337 (2)	C13—H13	0.9500
N1—C4	1.348 (2)	C14—H14	0.9500
N2—C1	1.332 (2)	N21—C21	1.160 (2)
N2—C2	1.3444 (19)	C21—S21	1.6320 (16)
N2—Ni1^ii^	2.1200 (13)	N31—C31	1.161 (2)
C1—H1	0.9500	C31—S31	1.6250 (16)
C2—C3	1.376 (2)	O41—C41	1.418 (2)
C2—H2	0.9500	O41—H1O4	0.80 (3)
C3—C4	1.377 (2)	C41—H41A	0.9800
C3—H3	0.9500	C41—H41B	0.9800
C4—H4	0.9500	C41—H41C	0.9800
			
N31—Ni1—N21	176.02 (6)	C4—C3—H3	121.0
N31—Ni1—O41	89.22 (6)	N1—C4—C3	121.66 (15)
N21—Ni1—O41	87.09 (5)	N1—C4—H4	119.2
N31—Ni1—N11	90.45 (5)	C3—C4—H4	119.2
N21—Ni1—N11	90.90 (6)	C14—N11—C11	117.02 (16)
O41—Ni1—N11	87.81 (5)	C14—N11—Ni1	122.48 (11)
N31—Ni1—N2^i^	87.56 (5)	C11—N11—Ni1	120.49 (12)
N21—Ni1—N2^i^	90.73 (5)	N11—C11—N11^iii^	125.2 (2)
O41—Ni1—N2^i^	86.62 (5)	N11—C11—H11	117.4
N11—Ni1—N2^i^	174.11 (5)	N11^iii^—C11—H11	117.4
N31—Ni1—N1	92.29 (5)	C14—C13—C14^iii^	117.1 (2)
N21—Ni1—N1	91.44 (5)	C14—C13—H13	121.4
O41—Ni1—N1	177.66 (5)	C14^iii^—C13—H13	121.4
N11—Ni1—N1	90.39 (5)	N11—C14—C13	121.79 (16)
N2^i^—Ni1—N1	95.23 (5)	N11—C14—H14	119.1
C1—N1—C4	116.23 (14)	C13—C14—H14	119.1
C1—N1—Ni1	122.94 (10)	C21—N21—Ni1	166.20 (14)
C4—N1—Ni1	120.79 (11)	N21—C21—S21	178.86 (16)
C1—N2—C2	117.01 (14)	C31—N31—Ni1	164.08 (13)
C1—N2—Ni1^ii^	122.90 (10)	N31—C31—S31	179.26 (16)
C2—N2—Ni1^ii^	119.50 (11)	C41—O41—Ni1	128.45 (12)
N2—C1—N1	125.96 (13)	C41—O41—H1O4	110 (2)
N2—C1—H1	117.0	Ni1—O41—H1O4	121.7 (19)
N1—C1—H1	117.0	O41—C41—H41A	109.5
N2—C2—C3	121.22 (15)	O41—C41—H41B	109.5
N2—C2—H2	119.4	H41A—C41—H41B	109.5
C3—C2—H2	119.4	O41—C41—H41C	109.5
C2—C3—C4	117.90 (14)	H41A—C41—H41C	109.5
C2—C3—H3	121.0	H41B—C41—H41C	109.5

Symmetry codes: (i) −*x*+1, −*y*+1/2, *z*+1/2; (ii) −*x*+1, −*y*+1/2, *z*−1/2; (iii) −*x*+3/2, −*y*+1/2, *z*.

### Hydrogen-bond geometry (Å, °)

**Table:**

*D*—H···*A*	*D*—H	H···*A*	*D*···*A*	*D*—H···*A*
O41—H1O4···S21^iv^	0.80 (3)	2.50 (3)	3.2474 (14)	154 (2)

Symmetry codes: (iv) *x*+1/4, −*y*+1/4, *z*+1/4.
